# Supplementary material for: Identification and Analysis of Aluminum-Activated Malate Transporter Gene Family Reveals Functional Diversification in Orchidaceae and the Expression Patterns of Dendrobium catenatum Aluminum-Activated Malate Transporters
Source: Int J Mol Sci. 2024 Sep 6;25(17):9662. doi: 10.3390/ijms25179662 (PMC11394931; doi:10.3390/ijms25179662)
Supplement: Supplementary file 1 [file ijms-25-09662-s001.zip › Supplement Figure.pdf]

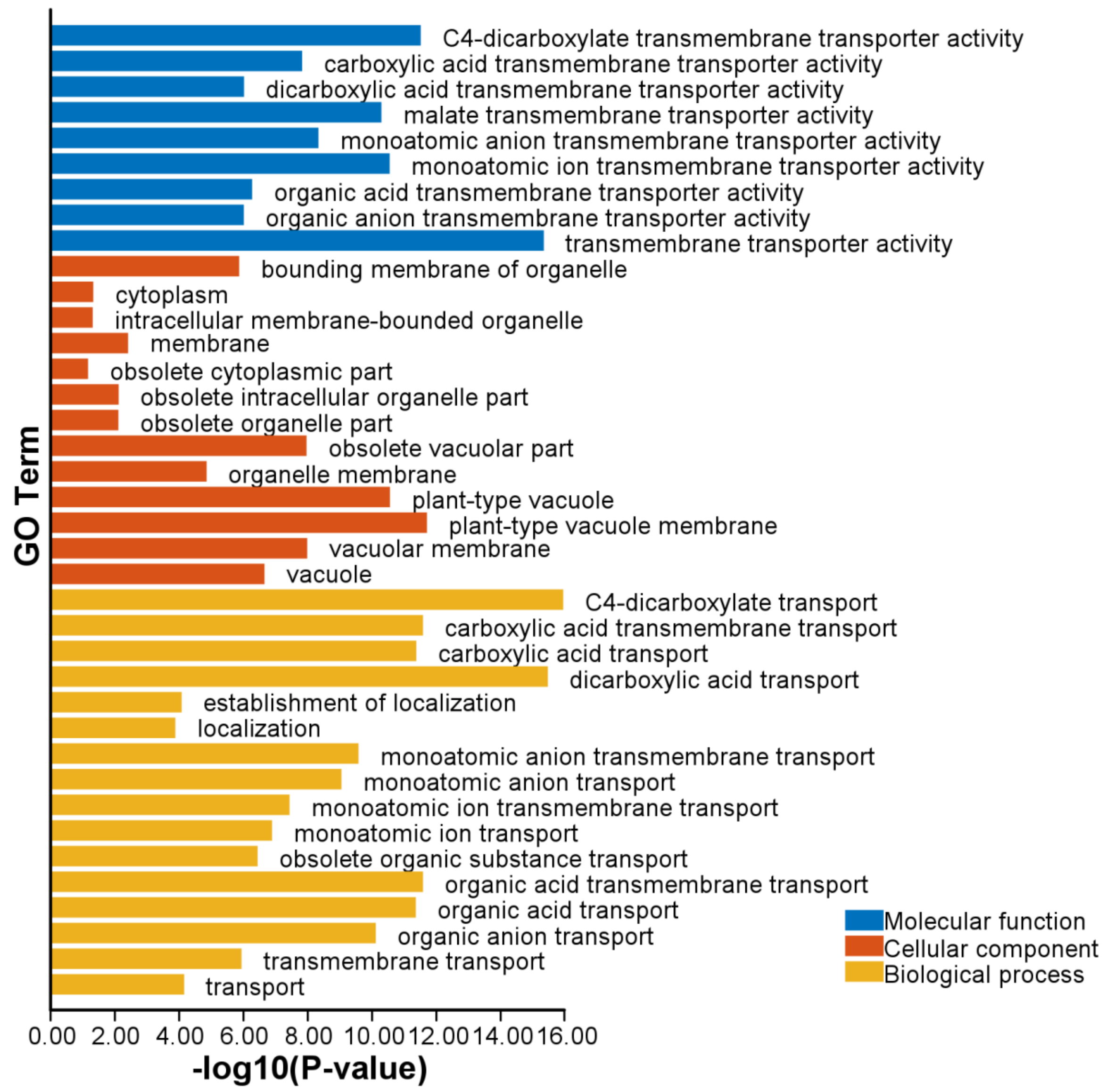

Figure S1. Gene ontology (Go) terms of orchid *ALMT* genes.

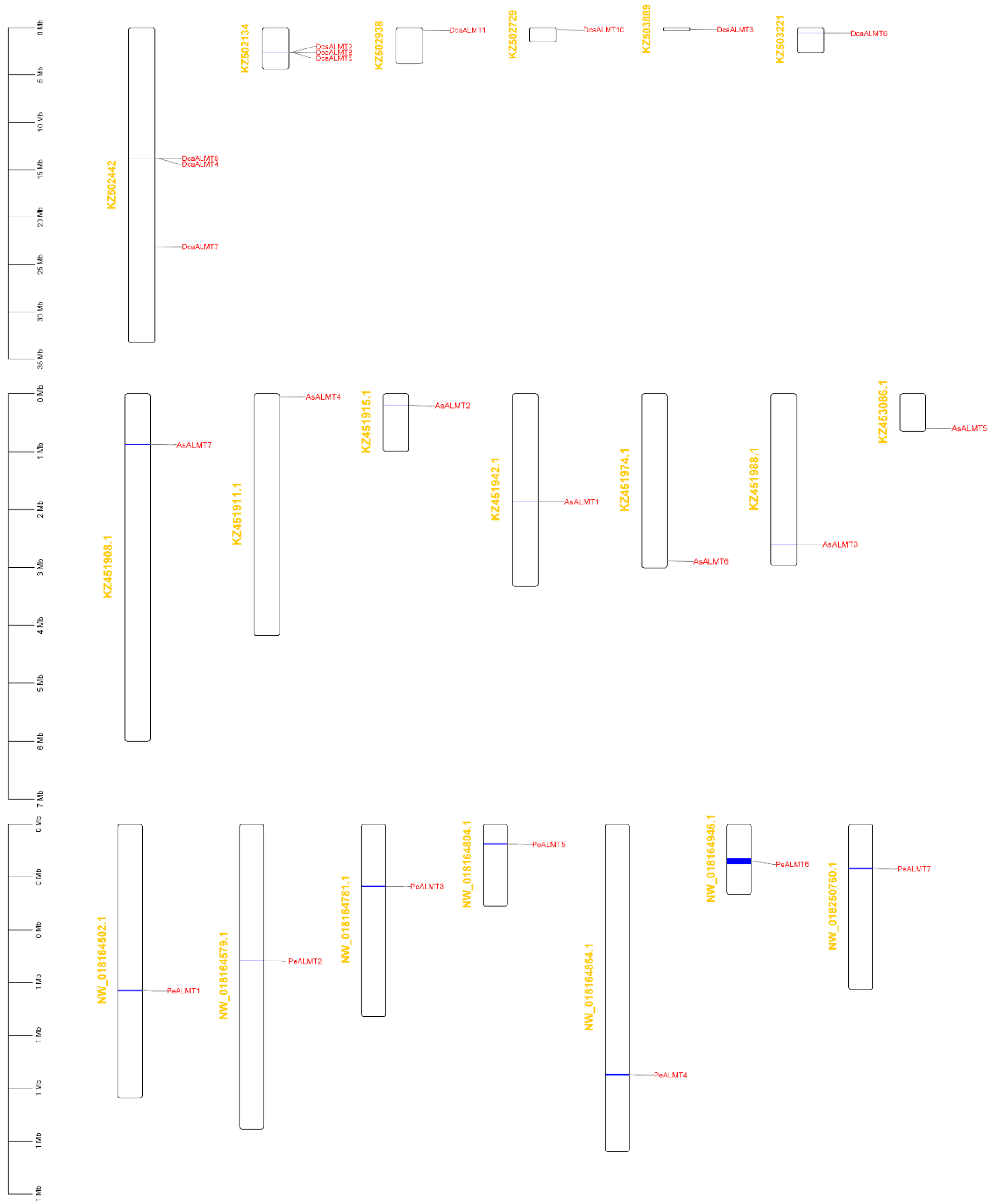

Figure S2. Scaffold localization of *ALMT* genes.

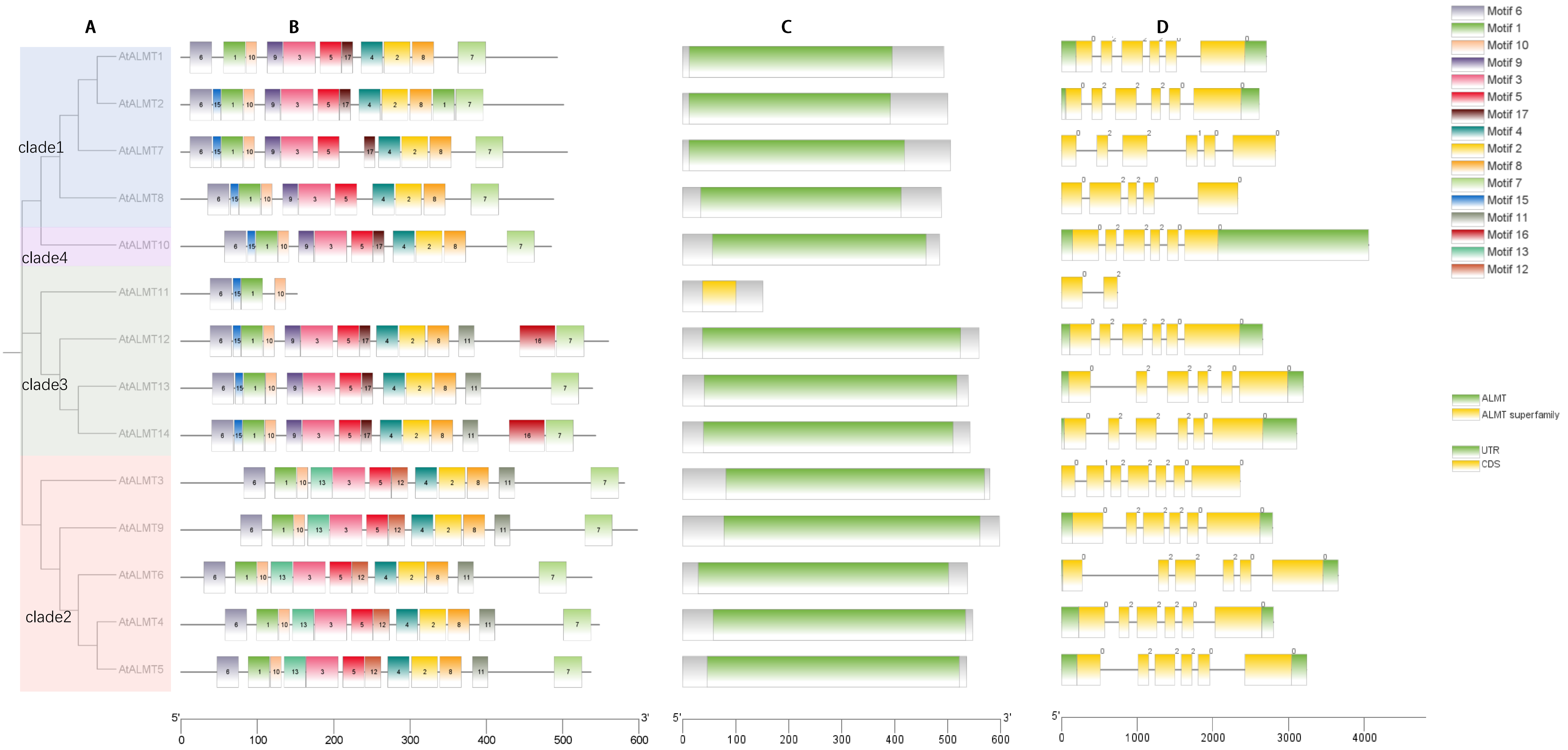

Figure S3. Gene structure, conserved motifs, and domains of AtALMTs.
